# Secondary Hemorrhage After Total Laparoscopic Hysterectomy: An Observational Study

**DOI:** 10.7759/cureus.79956

**Published:** 2025-03-03

**Authors:** Vibisha Pragash, Srikala D Reddy

**Affiliations:** 1 Obstetrics and Gynaecology, Apollo Hospitals, Chennai, IND

**Keywords:** cervical reasons, cervicitis, laparoscopic hysterectomy complications, obesity, secondary hemorrhage

## Abstract

Background

Secondary hemorrhage post total laparoscopic hysterectomy (TLH) is defined as bleeding per vagina after 24 hours up to 6 weeks after the primary surgery. The purpose of the study is to determine the possible risk factors for secondary hemorrhage after TLH and how to manage it medically.

Methods

A prospective observational study with all patients who underwent TLH under the author from 1^st^ January 2023 to 31^st^ December 2023, for indications requiring hysterectomy and suffering bleeding per vaginum starting from 24 hours until 6 weeks after the primary surgery were included in this study. Patients with a known history of bleeding disorders or those on oral/ parenteral anticoagulant medications were excluded.

Results

A total of 50 patients underwent TLH during the study period and 12 (24%) had secondary hemorrhage after hysterectomy. A significant p-value was noted in elevated BMI (0.037) and cervical pathology (0.036). There was found to be no significance in age, last childbirth, prior pelvic surgeries, pelvic adhesions, and other pelvic pathologies.

Conclusion

With more preference given to TLH than other modalities of hysterectomy, caution should be exercised regarding the potential risk factors. In patients with elevated BMI as well as cervical pathologies, necessary precautions should be taken as elaborated in the Discussion section.

## Introduction

Secondary hemorrhage post total laparoscopic hysterectomy (TLH) is defined as bleeding per vagina after 24 hours up to 6 weeks after the primary surgery. With the latest emerging laparoscopic instruments, the techniques of doing hysterectomy have tremendously reduced patient discomfort. Despite all this, the incidence of secondary hemorrhage post-TLH has been reported to be higher than in other modalities of hysterectomy [1,2]. Ten years back, a study conducted by Paul et al. reported an overall cumulative incidence of secondary hemorrhage after TLH of 1.3% (a retrospective study with 1613 patients over a period of 9 years) [2]. However, large populations could be challenging for close follow-up. Close follow-up was feasible in this study in view of small population size. In the initial days when laparoscopy was introduced, laparoscopic-assisted vaginal hysterectomy was preferred, but with the improved laparoscopic instruments and laparoscopic surgical techniques, TLH emerged and became a more preferred choice. In this study, the necessary preparation of the surgical site was done as elaborated in the methodology section. Intra-operatively from the pedicles to the uterine artery high-frequency ultrasonic vibrations (that is harmonic scalpel) were used followed by bipolar cautery for the uterine arteries and vaginal vault opening was done with the help of a monopolar hook. Our study showed secondary hemorrhage of 24% (12 patients) ranging from mild to moderate amount of bleeding. With TLH becoming more commonly used the incidence of secondary hemorrhage is also increasing. Therefore, creating awareness is becoming more than necessary. This awareness should include the potential risk factors that we should be cautious about. The awareness should also include how medical management of such patients can be done (with mild to moderate amounts of bleeding).

## Materials and methods

This is a prospective observational study where all patients who underwent TLH under the author at Apollo Hospital, Chennai from 1st January 2023 to 31st December 2023, for indications requiring hysterectomy and suffered bleeding per vaginum starting from 24 hours until 6 weeks after the primary surgery were included in this study. Patients with a known history of bleeding disorders or those on oral/ parenteral anticoagulant medications were excluded. The primary objective of this study is to analyze the possible link between risk factors such as vaginal vault infection, uterine size, past history of pelvic surgeries, pelvic adhesions, intra-operative adhesiolysis, BMI, and histopathological reports. A total of 50 patients met the eligibility criteria. The Institutional Ethics Committee-Biomedical Research, Apollo Hospitals, Chennai approved the study (Approval number: ASH-C-S-059/07-24).

Methodology

Fifty women who underwent TLH with informed signed consent and who fit the inclusion/exclusion criteria were included in this study. Within 6 weeks, 12 patients reported bleeding exceeding normal limits. Blood loss assessment involved counting the number of pads used per day, using a standard pad across various brands, estimating roughly 38.7 ml of blood [3]. The results were recorded in Excel format, followed by statistical analysis regarding their efficacy. The following data were analyzed: age, BMI, pre-operative hemoglobin (Hb), last childbirth (LCB), USG findings anteroposterior diameter (APD) and endometrial thickness (ET), history of cesarean section, past history of other pelvic surgeries, vault suture materials, indications for surgery, adhesiolysis, histopathological examination (HPE) impressions, and postoperative vaginal vault infections. For all patients pre-operatively chlorhexidine wash was provided, also betadine solution was used for painting the surgical field - with the necessary contact area and contact time provision. The intraoperative period was uneventful and blood loss was within normal limits. The postoperative period was uneventful. Proper surgical techniques were employed during TLH. The uterine arteries were coagulated using bipolar cautery with an average power of 35 - 45 Watts. Laparoscopic vaginal vault opening was performed with a monopolar hook (35 - 45 Watts of current) above the uterosacral arch, preserving it. The vaginal vault closures were completed using either barbed or non-barbed absorbable sutures.

All women adhered to our postoperative advice, which included avoiding physical exertion and refraining from sexual intercourse.

Statistical analysis

Sample Size

A prospective observational study from 1st January 2023 to 31st December 2023, under a single author, and the patients who fit the eligibility criteria were included. The statistical analysis methods are as described. Descriptive statistics were presented as mean ± SD and frequency (percentage) for the continuous and categorical factors, respectively. The median (IQR) was presented for skewed data. The normality of the data was assessed using the Shapiro-Wilk test. Student's t-test/Mann Whitney U test was employed to determine significant differences between bleeders and non-bleeders. Chi-square/Fisher’s exact test was applied to assess associations between two independent categorical factors. All analyses were performed using SPSS (IBM, 28.0, Armonk, USA).

## Results

In this study, The mean age of bleeders was 45.42 ± 6.89 years (range: 35-58), while non-bleeders had a mean age of 48.32 ± 8.40 years (range: 34-70). The difference was not statistically significant (p = 0.3). The time since last childbirth was comparable between the groups, with bleeders having a mean duration of 19.08 ± 7.94 years (range: 5-30) and non-bleeders 19.71 ± 8.59 years (range: 6-39) (p = 0.823). A notable finding with a significant p-value of 0.037, was noted in elevated BMI (overweight and obesity) among patients with secondary hemorrhage. Ten out of 12 patients (83.3%) had a BMI in the overweight or obese category, with 4 patients having a BMI >30. This suggests a potential correlation between higher BMI and an increased risk of secondary hemorrhage, though further studies are needed to establish causality (Table 1).

**Table 1:** Demographic Analysis of Age, Last Child Birth & BMI *Student’s t-test

Variable	Bleeders (Mean ± SD (Range)) (n=12)	Non-Bleeders (Mean ± SD (Range)) (n=38)	P-Value*
Age (In years)	45.42 ± 6.89 (35 – 58)	48.32 ± 8.40 (34 – 70)	0.3
Last childbirth (In years)	19.08 ± 7.94 (5 – 30)	19.71 ± 8.59 (6 – 39)	0.823
Body mass index (BMI)	28.77 ± 3.68 (21.90 – 35.40)	25.96 ± 4.04 (19.3 – 35.40)	0.037

A past history of pelvic surgeries that the patient had undergone was also noted. History of lower segment cesarean section (LSCS) was present in 6 (50%) of bleeders and 21 (55.3%) of non-bleeders (p > 0.99). Eight (66.7%) of bleeders and 26 (68.4%) of non-bleeders had a past history of other pelvic surgeries (p > 0.99). Adhesiolysis was found in 7 (58.3%) of bleeders and 24 (63.2%) of non-bleeders (p > 0.99). There was no significant difference between the groups in terms of prior surgeries (Table 2).

**Table 2:** Past History of Pelvic Surgeries and Their Outcomes LSCS: lower segment cesarean section ^#^Fisher’s exact test

Variables		(n=50), n (%)		P-Value^#^
		Bleeders, (n=12)	Non-Bleeders, (n=38)	
LSCS	Yes	6 (50%)	21 (55.3%)	>0.99
	No	6 (50%)	17 (44.7%)	
Other Pelvic Surgeries	Yes	8 (66.7%)	26 (68.4%)	>0.99
	No	4 (33.3%)	12 (31.6%)	
Adhesiolysis	Yes	7 (58.3%)	24 (63.2%)	>0.99
	No	5 (41.7%)	14 (36.8%)	

Among the 50 patients analyzed, 12 patients (24%) experienced mild to moderate secondary hemorrhage, with bleeding ranging from occasional spotting to 2-3 pads per day (approximately 77.4 - 116.1 ml) and lasting for an average duration of one week. Of these, 10 patients were managed conservatively with oral tranexamic acid (3-5 days) and prophylactic oral cephalosporins, while one patient required silver nitrate cauterization along with oral antibiotics and tranexamic acid due to unhealthy granulation tissues in the vaginal vault. One case of mild spotting resolved spontaneously without intervention.

Regarding antibiotic usage, Inj. cefoperazone + sulbactam (3 g followed by 1.5 g) was the most commonly administered parenteral antibiotic, used in 6 out of 12 patients (50%) with secondary hemorrhage and 17 out of 38 patients (44.7%) without hemorrhage. Other IV antibiotics, including Inj. cefuroxime (1.5 g) and Inj. cefotaxime (1 g), were used in a smaller proportion of patients. For discharge oral antibiotics, cefuroxime 500 mg (3-5 days) was prescribed to four patients with secondary hemorrhage, while cefotaxime 200mg (5 days) was given to 8 patients (Table 3).

**Table 3:** Antibiotics Used Intraoperatively and Postoperatively

Parenteral Antibiotics (Intravenous)	Patients With Secondary Hemorrhage (n=12)	Patients without Secondary Hemorrhage (n=38)
Inj. Cefoperazone + Sulbactam 3 g followed by 1.5 g	6	17
Inj. Cefoperazone + Sulbactam 3 g + Inj. Cefuroxime 1.5 g	1	1
Inj. Cefuroxime 1.5 g	2	16
Inj. Cefotaxime 1 g	3	4
Discharge antibiotics (Oral)		
Tablet Cefuroxime 500 mg × (3-5 days)	4	17
Tablet Cefotaxime 200 mg × 5 days	8	21

The indications for hysterectomy were analyzed, and the findings revealed that 58.33% of patients with secondary hemorrhage had adenomyotic changes in the uterus, which was more than 10% higher than in non-hemorrhagic patients (47.37%). Similarly, uterine fibroids were found in 50% of hemorrhagic patients, compared to 44.74% of non-hemorrhagic patients. Pre-surgical indications involving cervical pathologies were observed to be only in 16.67% of hemorrhagic patients and 18.42% of non-hemorrhagic patients, whereas endometrial pathologies were identified in only one non-hemorrhagic patient (2.63%) and none of the hemorrhagic patients. Ovarian pathologies were less prevalent among hemorrhagic patients (25%) compared to non-hemorrhagic patients (36.84%). Additionally, adnexal changes and endometriosis were only noted in non-hemorrhagic patients, at 10.53% and 7.89%, respectively. Among postmenopausal patients, endometriotic changes were present in 8.33% of hemorrhagic patients and 7.89% of non-hemorrhagic patients, while other postmenopausal pathologies were seen exclusively in 21.05% of non-hemorrhagic patients. Notably, pelvic adhesions were observed in 41.67% of hemorrhagic patients compared to 36.84% of non-hemorrhagic patients, suggesting a potential association with secondary hemorrhage (Table 4).

**Table 4:** Indications for Hysterectomy

Indications		Hemorrhagic Patients (12)	Non-Hemorrhagic Patients (38)
Uterine pathologies	Uterine adenomyotic changes	7 (58.33%)	18 (47.37%)
	Uterine fibroid	6 (50 %)	17 (44.74%)
	Cervical pathologies	2 (16.67%)	7 (18.42%)
	Endometrial pathologies	0	1 (2.63%)
	Ovarian pathology	3 (25%)	14 (36.84%)
	Adnexal changes	0	4 (10.53%)
	Endometriosis	0	3 (7.89%)
Postmenopausal patients	Postmenopausal – Endometriotic changes	1 (8.33%)	3 (7.89%)
	Postmenopausal – Other pathologies	0	8 (21.05%)
	Pelvic adhesions	5 (41.67%)	14 (36.84%)

However, the histopathological analysis (HPE) revealed that nearly 83.3% of the secondary hemorrhagic patients showed inflammatory signs of the cervix and nearly 91.6 % had some or other cervical problems. A significant p-value of 0.036 was noted in cervical pathologies (Table 5).

**Table 5:** HPE Impression HPE: histopathological analysis ^#^Chi-square

HPE Impression	Bleeders (n=12)	Non-Bleeders (n=38)	P-value^#^
Cervical pathology - Inflammatory signs of the cervix, pre-cancerous lesions, and other cervical lesions	11 (91.7%)	21 (55.3%)	0.036
Adenomyotic changes	6 (50%)	19 (50%)	>0.99
Leiomyoma	6 (50%)	18 (47.4%)	>0.99
Other uterine pathologies – Endometrial changes, etc.	2 (16.7%)	12 (31.6%)	0.468
Adnexal pathology - Hematosalpinx, hydrosalpinx, salpingitis, etc.	6 (50%)	18 (47.4%)	>0.99
Ovarian pathology	1 (8.3%)	13 (34.2%)	0.140

The analysis of vault suture materials used in hysterectomy cases revealed no significant association between the type of suture material and the occurrence of secondary hemorrhage (p = 0.849). Among the 50 patients, the most commonly used suture was symmetric PDS plus (polydioxanone - STRATAFIX), which was used in 8 (66.7%) of bleeders and 27 (71.1%) of non-bleeders. VICRYL was utilized in 2 (16.7%) of bleeders and 7 (18.4%) of non-bleeders, while V-LOCTM 180 was used in 2 (16.7%) of bleeders and 4 (10.5%) of non-bleeders. The comparison between barbed (V-LOCTM 180, STRATAFIX) and non-barbed (VICRYL) sutures showed no statistically significant difference in the incidence of secondary hemorrhage, indicating that the choice of suture material did not have a notable impact on post-hysterectomy bleeding outcomes (Table 6).

**Table 6:** Types of Vault Suture Materials Used ^#^Chi-square

Vault Suture Materials	(n=50), n (%)		P-Value^#^
	Bleeders (n=12)	Non-bleeders (n=38)	
VICRYL	2 (16.7%)	7 (18.4%)	0.849
V-LOC^TM^ 180	2 (16.7%)	4 (10.5%)	
Symmetric PDS plus (Polydioxanone - STRATAFIX)	8 (66.7%)	27 (71.1%)	

The pre-surgical ultrasound findings demonstrated that the APD and ET were higher in bleeders compared to non-bleeders. The mean APD was 5.34 ± 1.26 cm in bleeders, compared to 4.74 ± 1.49 cm in non-bleeders, with a range of 3.20 - 7.40 cm and 2.39 - 9.10 cm, respectively. Similarly, the mean ET was 9.10 ± 2.97 mm in bleeders and 7.41 ± 3.71 mm in non-bleeders, with a range of 3 - 14 mm and 2 - 17.30 mm, respectively. Despite the observed increase in APD and ET values in patients with secondary hemorrhage, the difference did not reach statistical significance (p = 0.221 for APD and p = 0.160 for ET), indicating that these sonographic parameters alone may not be predictive markers for secondary hemorrhage (Table 7).

**Table 7:** Pre-operative ultrasound findings regarding anteroposterior diameter and endometrial thickness APD: Anteroposterior diameter; ET: Endometrial thickness *Student’s t-test

Ultrasound Findings	Bleeders (Mean ± SD (Range)) (n=12)	Non-Bleeders (Mean ± SD (Range)) (n=38)	P-Value*
APD (In cm)	5.34 ± 1.26 (3.20 – 7.40)	4.74 ± 1.49 (2.39 – 9.10)	0.221
ET (In mm)	9.10 ± 2.97 (3 – 14)	7.41 ± 3.71 (2 – 17.30)	0.160

## Discussion

Elevated BMI and cervical pathology were found to be potential risk factors for secondary hemorrhage after TLH with p-values <0.05. Age, prior pelvic surgeries, ultrasound findings (APD, ET), pelvic adhesions, and adhesiolysis do not seem to have any significance between bleeders and non-bleeders. This study did show to have a higher incidence of bleeders as compared to other previous studies/works of literature [2,4,5]. The reason could be because of the small population study (allowing close follow-up of the patients) despite all the patients being hemorrhagic or non-hemorrhagic did follow our postoperative medications and reported to the OP department with complaints of bleeding per vaginum or on the expected dates of review. Other studies may have had a lower incidence of secondary hemorrhage in view of the failure to report or non-documentation of the patients who presented with secondary hemorrhage. As stated by Paul et al., our study could have a higher incidence due to the preservation of the arch of uterosacral ligaments, which includes more cervical tissues and therefore have a greater chance of bleeding [2]. Adenomyotic changes do have an impact on the bleeding. This could be because of increased vascularity associated with adenomyosis. Although only 2 among the 12 bleeders had cervical pathology as an indication of hysterectomy, the final histopathological report depicted nearly 91.6% of the bleeders had some form of cervical pathology. Therefore, even the quiescent cervical lesions should be taken into consideration and these patients should be followed up with great caution. Many studies have proven that obese people have more complications post-surgery and also have denoted that obese patients could be treated with antibiotics which will also be as effective as in normal-weighing patients [6,7]. In our study, ultrasonic energy was used for coagulating and cutting cornual and para-uterine tissues. Bipolar cautery was used for uterine vessels. A monopolar hook was used for vault opening with due caution to not cause excessive thermal injuries to the vaginal vault [2]. Barbed vs. non-barbed vault suturing proved to have no difference as shown in Table 6. It only proved to be time conserving on vault suturing as proved in multiple literatures [8-11]. During the surgical procedure of TLH, the blood vessels were thoroughly coagulated, but in view of pelvic viscera being supplied by anastomotic blood vessels, the chances of control of bleeding can be limited especially if the bleeding site is extensive or not identifiable. There may be circumstances where the bleeding may not be anatomically accessible. Patel et al. stated that inflammatory reactions including pelvic adhesions are also responsible for the failure of surgical explorations which however did prove to be contradictory in our study [12]. However, cervical pathologies, including inflammatory signs of the cervix (cervicitis), do seem to pose a risk of secondary hemorrhage after TLH. Vaginal vault infection (9% after laparoscopic hysterectomy as shown by Clarke-Pearson and Geller) is one of the risk factors for secondary hemorrhage [13]. The patient in this study had no indications suggestive of vaginal vault infection. However, India has a hot and humid climate favorable for bacterial growth, and hygiene maintenance of genital regions could be difficult, that is why an initial prophylactic short course of oral antibiotics was provided. Also, studies have shown that people with higher BMI are more prone to infection [14-17]. Semins et al.’ s study showed that women were at a higher risk of developing infections [17]. The limitations of this study could be a small population size, where the exact cause of secondary hemorrhage could not be completely analyzed but only certain risk factors could be analyzed. This limitation can be overcome by conducting a large multicentric study. However, despite the exact cause of secondary hemorrhage is not yet known it is still proven that compared to laparotomy, laparoscopic hysterectomy is still beneficial to patients as they recover faster and have a comparatively lower morbidity. Also, larger multicenter studies to strengthen the findings would be needed.

## Conclusions

Despite secondary hemorrhage post-TLH being rare, our study showed 24% occurrence, and all the patients were managed medically and didn’t need surgical intervention. Our study showed elevated BMI (overweight/obesity) and cervical pathologies are potential risk factors for secondary hemorrhage after TLH.
